# Morphological Development of Sub-Grain Cellular/Bands Microstructures in Selective Laser Melting

**DOI:** 10.3390/ma12081204

**Published:** 2019-04-12

**Authors:** Xihe Liu, Xin Zhou, Ben Xu, Jing Ma, Congcong Zhao, Zhijian Shen, Wei Liu

**Affiliations:** 1 School of Materials Science and Engineering, Tsinghua University, Beijing 100084, China; liuxihe.pku@gmail.com (X.L.); xuben@mail.tsinghua.edu.cn (B.X.); ma-jing@mail.tsinghua.edu.cn (J.M.); zhaocc819@mail.tsinghua.edu.cn (C.Z.); zhijian.james.shen@mmk.su.se (Z.S.); 2 Science and Technology on Plasma Dynamics Laboratory, Air Force Engineering University, Xi’an 710038, China; 3 Department of Materials and Environmental Chemistry, Arrhenius Laboratory, Stockholm University, S-106 91 Stockholm, Sweden

**Keywords:** selective laser melting, thermocapillary convection, Bénard-Marangoni-instability, rapid solidification, sub-grain microstructures

## Abstract

In this paper, single-layer and bulk 316 L selective laser melting (SLM) experiments were conducted, fine submicron-scale geometric symmetrical cellular (hexagonal, pentagonal and square), elongated cellular and bands solidification morphologies were found in the laser-melt top surface. Meanwhile, morphological developed sub-grain patterns with quasi-hexagonal cellular, elongated cellular and bands structures (size ~1 μm) coexisting inside one single macro-solidified grain were also identified. This demonstrated the transitions from quasi-hexagonal-cells to elongated cells/bands, and transitions reverse, occurred in the whole bulk under some circumstances during SLM. Based on the experimental realities, these morphologies are formed by the local convection and Bénard instabilities in front of the solid/liquid interface (so-called mushy zones) affected by intricate temperature and surface tension gradients. Quasi-hexagonal cellular convective fields are then superimposed on macro-grain solidification to form the sub-grain patterns and micro-segregations. This explanation seems reasonable and is unifying as it can be expanded to other eutectic alloys with face center cubic (FCC) prevenient phase prepared by SLM, e.g., the Al-Si and Co-Cr-Mo systems.

## 1. Introduction

Selective laser melting (SLM) is a member of the additive manufacturing family of technologies whereby a three-dimensional (3D) part is built layer by layer by laser scanning of a precursor powder bed [1,2]. The physical feature of SLM is very similar to the micro-beam laser welding, both processes involve the fusion of the material with rapidly scanning of a small heat source [3]. This is a system out of thermodynamic equilibrium, several instabilities happen simultaneously and the non-equilibrium solidification, multiscale hierarchical SLM microstructures will respond differently than conventional processing technologies [4,5].

The typical characteristics of SLM alloys microstructures are the unique sub-grain patterns. These sub-grain (0.5~1 μm) cellular/bands structures were found inside each individual large grain in SLM stainless steel [6,7,8,9,10,11], molybdenum was found to be enriched at the 316 L austenite sub-grain boundaries [6]. Besides stainless steel, Al-Si is another alloy system which can form the unique sub-grain microstructures [12,13,14,15,16,17,18,19], fine supersaturated Al-rich cellular structures along with Si micro-segregations at the boundaries are very common to see. In addition, these morphologies can also be found in some other SLM alloys which are characterized by eutectic systems with FCC prevenient phase, e.g., Ni625 (Ni-Cr21.5-Mo9-Nb3.6, large amounts of Nb and Mo concentrated at cellular boundaries) [20], cell boundaries enriched in Cr, Mo but Co depleted (Co–Cr–Mo) [21,22], CoCrW [23], NiCr [24]. These fine sub-grain microstructures and fine distribution of particular elements are confirmed beneficial to the alloy’s hardness, strength, ductility (known as defect-tolerant) [14,25], and thermal conductivity performance under some circumstances [26]. This kind dynamical formation of three-dimensional arrays of cells and bands structures are also very common in high energy beam material processing and rapid solidification, e.g., welding [27,28,29,30], surface melting [31,32,33], space directional solidification [34,35], laser cladding or laser engineered shaping (LENSTM) [36,37,38,39,40]. These technologies have common features with SLM of constrained crystal growth, so the similar microstructures can be fabricated. However, these sub-grain patterns are found to only appear in some FCC alloys, but were not present in SLM Ti-6Al-4V (HCP), Fe (BCC) and ferrous alloy metal matrix composites [5]. When adding 10 wt.% Mo powder to Ti-6Al-4V, it changes from planar to cellular mode and the new cellular β grains (5–15 μm) are significant smaller than the earlier grains (50–150 μm). Meanwhile, within each β grain a cellular substructure with an intercellular spacing of less than 1 μm, presents and microsegregation of the elements Mo, Al and V takes place [41]. With all the discovered experimental phenomena, one can find these sub-grain microstructures are controlled by not only the processing thermodynamic conditions (temperature gradients and growth velocities), but also alloy compositions and even phase crystal structures, which need further understanding.

Some researchers present the constitutional supercooling and columnar to equiaxed transition (CET) theory, the ratio of temperature gradients and growth rates (G/R) decides the grain growth morphologies, heat accumulation may also provide the opportunity for a transition from columnar to equiaxed transition [10,40,42,43]. Some others employ the interface stability theory, which can be described as a periodic breakdown between dendritic (or eutectic) and plane front growth, by considering the undercooling of the solid-liquid interface as a function of the growth rate, the width inversely proportional to temperature gradients and growth rates [44,45,46,47]. Fukumoto and Kurz [30,48,49] also develop a solidification phase and microstructure selection map for Fe-Cr-Ni alloys, the different phase (austenite or ferrite) and different microstructure (eutectic, dendrite, band, plane front) are controlled by Cr/Ni ratio and growth velocity, theoretical predictions are compared with experimental results. But these explanations neglect the convection and Marangoni effect, then another researcher believed the source of unique patterns is assumed to be a convective or diffusive transport of impurities or one of the constituents of the material [50], fluid flow along a solid–liquid interface induces element segregation and morphological instabilities, the singularity of the highest solute concentration is the cause of instability [51,52]. SLM belongs to the system out of thermodynamic equilibrium, solid–liquid interface undergoes morphological instability while the melt is suffering hydrodynamic instability. Constitutional supercooling, interface instability and convective solute enrichment take place simultaneously, but the constitutional supercooling theory is based on thermodynamic equilibrium analysis, the non-equilibrium effects are not coupled, and the dynamic interfacial tension effects are neglected. In addition, the sub-grain structures in one single macro-grain could have variable geometric symmetrical patterns, e.g., hexagonal cellular, pentagonal cellular, square cellular, elongated cellular and bands/stripes, the plane-to-cell transition and interface instability are not just depend on linear stability limit and local equilibrium assumption, it is a non-linear process which need full coupled consideration of convective instability, interface morphological instability and surface-tension effects.

To get a further understanding of the forming mechanism, single-layer and bulk 316 L SLM experiments were designed and conducted in this study, the phenomenon of transitions from quasi-hexagonal-cells to elongated cells/bands in one macro-grain are identified; the relations between surface-tension-driven conventions, as-melted top surface geometric symmetrical morphologies and the sub-grain cellular/bands microstructures are established.

## 2. Materials and Methods

### 2.1. Material

We used 316 L stainless steel powder granules with an overall chemical composition of 17 wt.% Cr, 10.6 wt.% Ni, 2.3 wt.% Mo, 0.98 wt.% Mn, 0.4 wt.% Si, trace amounts of S, C, P, O, N and the balance being Fe as precursors, supplied by Sandvik Osprey Ltd., Neath, UK [6]. The powder granules were spherical with particle size of 22–53 μm determined by a laser diffraction analyzer (Mastersizer 2000, Malvern Instruments, Worcestershire, UK). Before the experiments, the powder’s granules were sieved (50 μm) under argon to reduce the agglomeration and to improve fluidity.

### 2.2. Experiment Arrangement and Procedures

All SLM experiments were conducted on a Renishaw AM250 facility equipped with a SPI redPOWER 200W ytterbium fiber laser, operating at 1071 nm wavelength and 75 μm beam diameter (Φ 99%) (Renishaw AMPD, Stone, UK). The laser ran in modulated operation (pulsed with transistor-transistor logic trigger).

**Figure 1:**
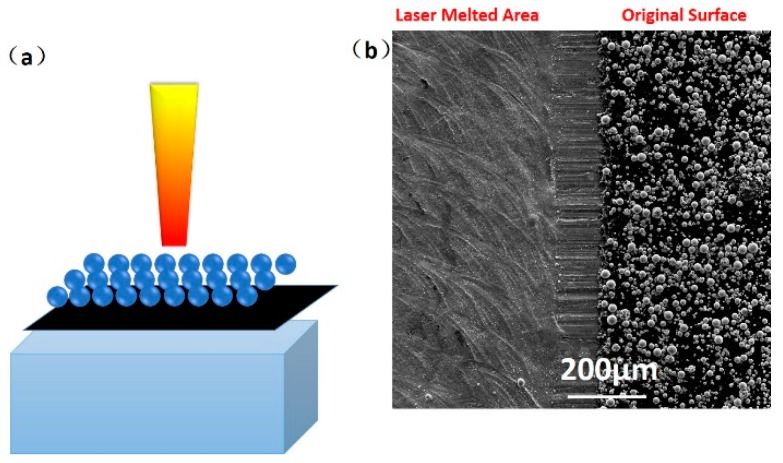
An illustration of the single-layer powder selective laser melting (SLM) experiment (**a**). The top surface appearance after and before laser scanning is shown by a divided scanning electron microscope (SEM) image with left and right areas, respectively (**b**).

Single-layer SLM tests were performed to study the as-melted top surface morphologies for the reason of ultra-high temperature/surface tension gradients it can generate, where the substrate was a rolled 316 L plate, size of 10 cm × 8 cm × 3 cm, polished with 1000# abrasive paper. The polished surface was then coated with a black paint layer to reduce the reflection of laser energy. The powder layer was deposited on the black painted surface with a thickness of 50 μm using the wiper system on the SLM machine, Figure 1a. The laser beam then scanned the single powder layer with power 190 W, scan speed 700 mm/s, line spacing 0.05 mm, and “zigzag” scan strategy. Oxygen content in the chamber was set to 1000 ppm. The single-layer powder appearance and laser-melt morphologies are shown in Figure 1b.

The 316L SLM bulks were fabricated using the same laser parameters as in the single-layer SLM experiments, except line spacing was 0.125 mm. The scan strategy was “cross hatching” which had long bi-directional scanning vectors and performed 67° angle rotation of scanning direction between adjacent layers.

### 2.3. Microstructural Characterization

Samples were microscopically characterized using a TESCAN MIRA 3LMH scanning electron microscope (SEM) from TESCAN (Brno, Czech Republic). The SEM samples were ground using sand paper in a Buehler abrasive belt grinder and followed by polishing with a set of decreasing diamond size suspensions with a final 1 μm size. Chemically etching occurred in an acidic water solution containing 2% HF-8% HNO_3_-deionized water for, 10 min at 25 °C. Transmission electron microscope (TEM) tests were performed on a JEM-2100 (JEOL, Tokyo, Japan). The TEM samples were first ground down to 70 μm thickness and then twin-jet electropolished to electron transparency; electrolyte was 10% perchloric acid in methanol and the temperature was maintained at −30 °C.

## 3. Results

### 3.1. The Top Surface Morphologies of Single-Layer Laser Melting

**Figure 2:**
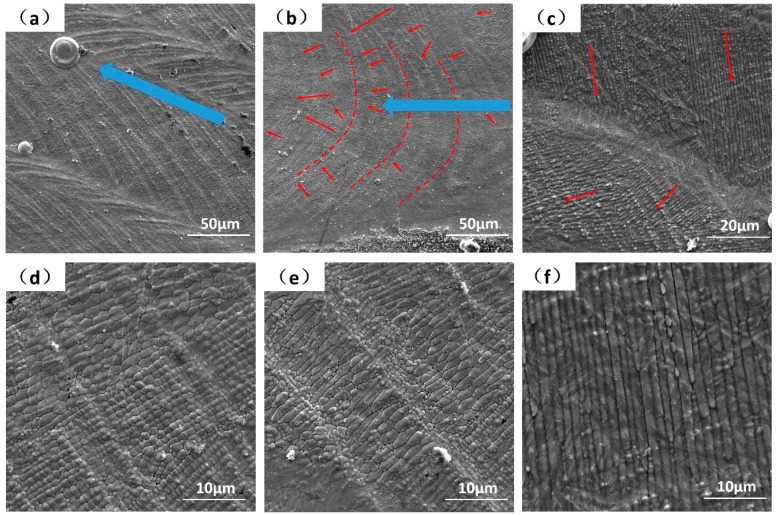
Top views of the single-layer powder laser melted top surface, observed at SEM with 1000× (**a**,**b**) and 2000× (**c**) magnification. The blue arrows demonstrate the scanning direction. The red dashed lines demonstrate the laser melt pool boundaries, the red arrows demonstrate the complex melt flow directions; Mixed hexagonal, pentagonal and square cellular patterns are shown in (**d**), elongated drifting cellular patterns in (**e**) and strip flow patterns in (**f**), observed with 5000×.

**Figure 3:**
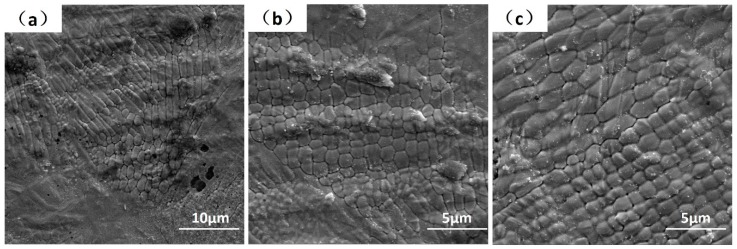
SEM images showing the geometric symmetrical cellular patterns in detail, demonstrating the mixed hexagon, pentagon and quadrilateral in the images (**a**–**c**), respectively.

Clear melt tracks and surface ripples can be observed at the single-layer laser melting surface, as seen in Figure 2a. These features are induced by the melt pool dynamics/oscillations and are typical surface phenomena observed in both welding and SLM [53]. More subtle solidification patterns can be found simultaneously, as cells and strips with different directions, but the main direction is always pointing to the melt pool center, see Figure 2b,c. These microscopic melt-solidified morphologies are also found as geometric symmetrical, three particular patterns can be distinguished by SEM at 5000× magnification. The first mode shows the mixed stationary hexagonal, pentagonal and square cellular patterns where the cellular size is only around 1 μm, see Figure 2d. More specific demonstrations of these geometric symmetrical cellular patterns are shown in Figure 3, with hexagon (Figure 3a), pentagon (Figure 3b) and quadrilateral (Figure 3c). The second mode displays a “drifting cell”, which can be considered as an elongated hexagonal cell, see Figure 2e and Figure 3a. Finally, the third mode shows the appearance of “long strips” that have a width of only 1 μm, but with a length over 50 μm, see Figure 2f. The forming mechanism of these three as-melted top surface patterns can be explained by the interaction between convection instabilities in front of the solidification front and solute transport behavior, which will be further discussed in Section 4 below.

### 3.2. The Sub-Grain Cellular/Bands Microstructures

**Figure 4:**
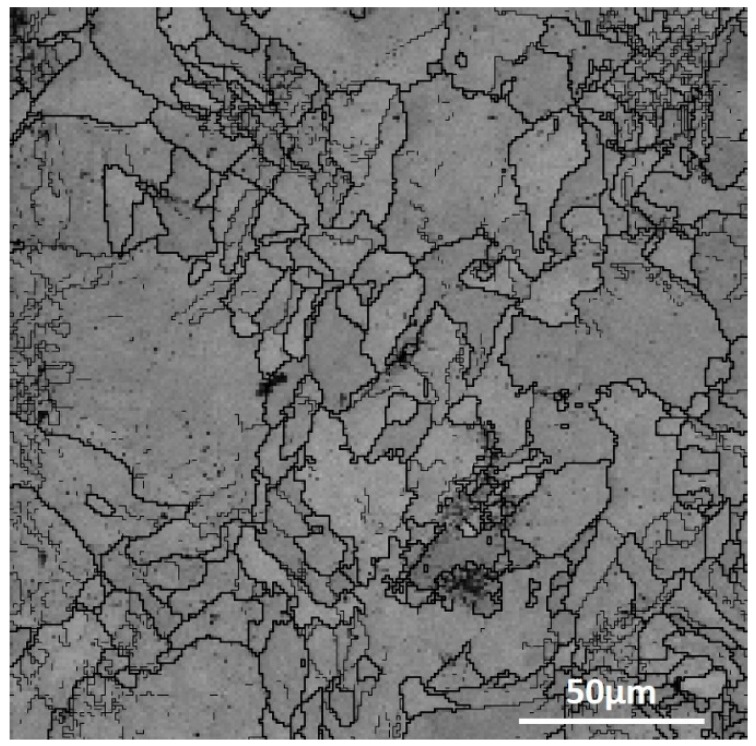
The high-angle grain boundaries were analyzed by electron backscattered diffraction (EBSD).

The solidified macro-grain boundaries (classified by grain orientations) in SLM 316 L stainless steel are shown in Figure 4. As mentioned in [6], 316 L SLM solidifies as full austenite, probably for the very high growth velocity [30]. The size distributions of these irregular austenitic macro-grains were not uniform, there were both larger grains with size over 50 μm and smaller grains, but the median macro-grain size according to the graphical analysis results was around 10 μm.

**Figure 5:**
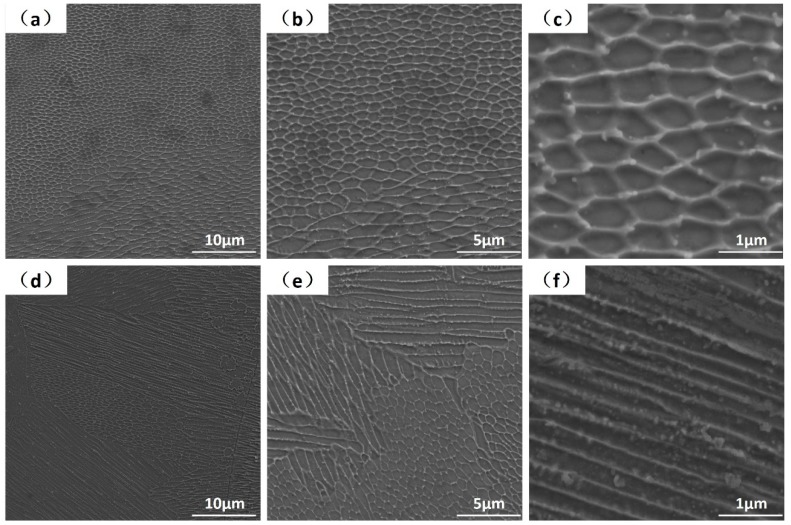
The SEM images of a variety of sub-grain microstructures; with mixed cellular and band morphologies (**a**–**f**). All the images were exposed upon a transverse cross-section normal to the building direction.

The macro-grains had very complex substructures and SEM images from polished and chemically etched surfaces are given as examples in Figure 5. Distinct and complex fine band/cellular sub-grain microstructures were revealed. There exist cellular and elongated cellular sub-grain patterns illustrated in Figure 5a–c. These are analogous in sizes and shapes to the as-melted top surface hexagonal patterns shown in Figure 3. At the same time, the band structures in Figure 5d–f are analogous to the as-melted top surface strip patterns shown in Figure 2f. Morphological developed quasi-hexagonal-cellular and elongated cellular structures appeared simultaneously, as highlighted in Figure 5b. This demonstrates the transitions from quasi-hexagonal-cells to elongated cells are natural under some circumstances. Furthermore, since transition from cellular to strip patterns and even strips crossover can also be found in Figure 5e, means that it was not the dendrite structure as understood with different observation from transverse or vertical cross section, as talked about in [22]. The band structures were formed in the following sequence: quasi-hexagonal-cells → elongated cells → bands. These transitions were very confusing as they can exist in one single macro-grain and occur in the whole bulk. Current theoretical explanations of constitutional supercooling, columnar to equiaxed transition (CET) and lateral instabilities beneficial to the growth of secondary arms are all not very convincing. Therefore, we suggest that these observations are nonlinear self-organization phenomena under the strong marangoni convection in front of the S/L interface, as proved and discussed in detail below.

### 3.3. The TEM Observation

**Figure 6:**
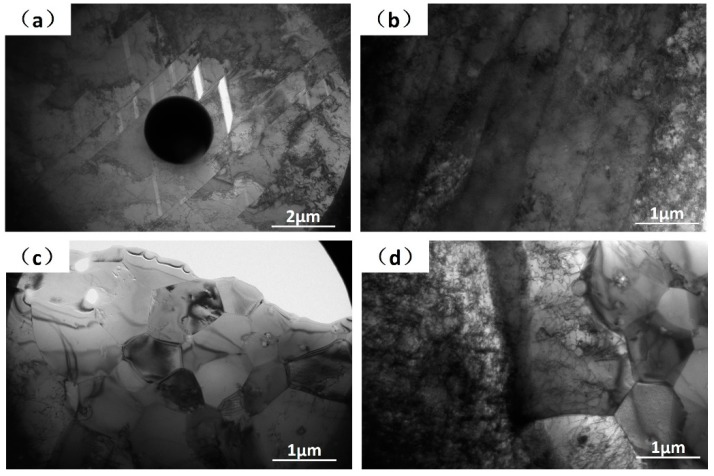
Transmission electron microscope (TEM) images are shown from band (**a**,**b**) and cellular microstructures (**c**,**d**). The black circular inclusion in (a) is a Cr–Si–O particle.

Sub-grain bands and cellular microstructures can be distinguished by TEM images, as seen in Figure 6. The black circular inclusion in Figure 6a is an amorphous Cr–Si–O particle which has been discussed in [6]. Clearly dislocation tangles were found in the TEM images, but the dislocations were not homogeneously distributed in the material, as in some area the density was very high while in other areas it was absent. Because the SLM samples were not processed by plastic deformation, so the sub-grain patterns were not the dislocation or twinning structures. Actually, these are solidification sub-grains which may relate to the asymmetry of temperature gradients, impurity segregations and possible solid-state phase change of ferrite to austenite during rapid cooling.

## 4. Discussion

### 4.1. The Complex Thermocapillary and Solutocapillary Convections in the Melt Pool

**Figure 7:**
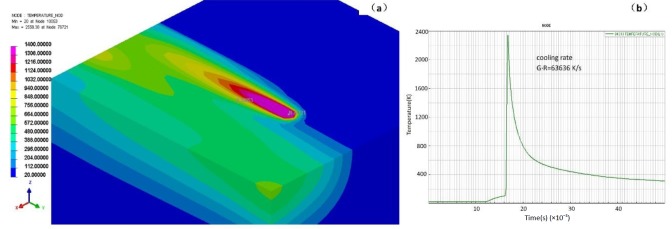
FEM results of 316 L by SLM; isometric view (**a**) and plot of node temperature vs. time relationship (**b**). The cooling rate (G·R, K/s) can be calculated.

**Figure 8:**
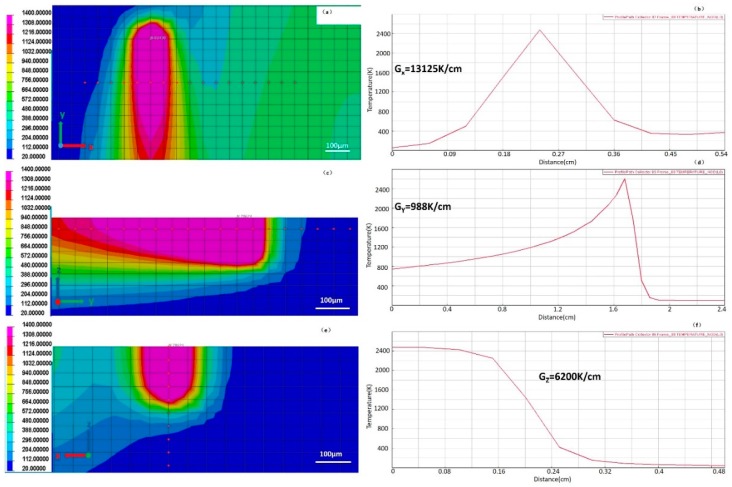
FEM results of 316 L by SLM. The melt pool by a top view (**a**), a longitudinal-section view (**c**) and a cross-section view (**e**), where a plot of node temperature vs. distance can be found in (**b**), (**d**) and (**f**), respectively. The involved nodes are also marked separately in (a), (c) and (e). Temperature gradients of the three different directions (G_x_, G_y_, G_z_) can be calculated, where G_x_ represents the temperature gradient in the top surface melt pool edge, G_y_ represent the gradient in the melt pool tail and G_z_ represent the gradient in the melt pool bottom.

The simulation was carried on SYSWELD 2010, the results were calculated out of a heat transfer model under the effect of a Gaussian heat resource. The processing parameters in the simulations were the same as those in the experiments. The SLM technique comprised of very complex physical processes, finite element simulation of the SLM process used on 316 L steel can be seen in Figure 7 and Figure 8 [54]. A finger shaped melt pool formed at ultra-high heating/cooling rates when laser irradiates the surface in a straight line. The node temperature increased rapidly to a maximum around 2400 K in Figure 7. After the laser moved away the node temperature dropped rapidly and the cooling rate can be estimated to around 6 × 10^4^ K/s. The temperature gradients in different cross profiles were found in Figure 8. The width of the elongated melt track was around 150 μm, but the length was near 1 mm. A high temperature gradient existed at the melt track edge perpendicular to laser moving direction, in Figure 8a. The value of G_x_ can be calculated as high as 1.3 × 10^4^ K/cm, see Figure 8b. A longitudinal-section view revealed that the melt had a maximum depth of 200 μm and a tail with gradual reduced depth, in Figure 8c. The temperature gradient G_y_ along the tail was only 1.0 × 10^3^ K/cm, see Figure 8d. At a cross-section view, the temperature gradient G_z_ in the melt pool bottom (perpendicular to laser scan surface) was about 6.2 × 10^3^ K/cm, in Figure 8e,f.

The mentioned non-uniform temperature gradients found in the melt pool can induce surface tension variations and generate thermocapillary flow. The direction of a thermocapillary flow depends on the temperature coefficient of the surface tension, *∂*σ/*∂*T. In addition, inhomogeneity of surface tensions may also result from concentration variations of other solutes altering the surface energies, e.g., dissolved surface-tension active components (S, O) and element enrichments of a multi-element alloy. Surface tension is a function of temperature *T* and the contents of other elements [55]:

(1) $$\frac{\partial \sigma }{\partial T}=-A-R\Gamma_{s}\ln(1+K_{\mathrm{seg}}a_{i})-\frac{K_{\mathrm{seg}}a_{i}}{1+K_{\mathrm{seg}}a_{i}}\frac{\Gamma_{s}\Delta H^{0}}{T}$$

where *∂*σ/*∂*T is the coefficient (temperature and surface-active elements) of surface tension; *R* is the gas constant; Γ*_s_* is the saturated surface excess; K_seq_ is the equilibrium absorption coefficient of surface-active elements; a_i_ is the activity of surface-active elements (weight %); and Δ*H*^0^ is the standard heat of adsorption.

In our experiments, although the SLM chamber environment was carefully controlled, it was still difficult to remove all surface-active elements completely, e.g., oxygen. Some residual oxygen was present in the precursor powder and in the SLM machine chamber (<1000 ppm). In the SLM process, the center of laser melt pool had the highest temperature and as ∂σ/*∂*T was a negative value, the melt at the top surface flowed outward (Equation (1)). In the edge area, with significant lower temperature, the *∂*σ/*∂*T changed to a positive value and the melt flow inverted inward from the edge to the center. The SEM images presented in Figure 2 can be understood by this mechanism. Firstly, at the melt pool edge the largest temperature gradients exist with a dissolved surface-active element film, resulting in the described melt flow from edge to the center. Secondly, at an invariable cooling rate of 6 × 10^4^ K/s (G·R) and the smallest temperature gradient being along the tail (1.0 × 10^3^ K/cm), the growth rate R had a maximum value of 600 mm/s (laser speed 700 mm/s) from the tail to the center. Most important is that these ultra-high, nonlinear and asymmetrical temperature gradients can initiate intense melt jets and even turbulence instabilities, with surface flow rates higher than 1000 mm/s [56], which will then form complicated flow patterns and solidification microstructures. Furthermore, the element enrichment in front of the S/L interface can also influence the thermocapillary flow mode, two examples are the rejected elements of Si, Cr, and Mo during austenitic solidification in 316 L and the precipitation of Si phase in the Al–Si eutectic system, these elements can change the local surface tension gradients.

### 4.2. Bénard Instabilities and Cellular Microstructures in Front of the S/L Interface

The SLM process itself raises some specific conditions, the size of a SLM melt pool was limited, width and depth are around 100~200 μm, and the process can be treated as a mesoscale film flow in which the gravity effect can be ignored, and surface forces are greater than volume forces. The melt pool had extreme temperature gradients (~10,000 K/mm), inevitable giving intense convection and turbulent heat- and mass-transfers [57]. The hexagonal-cellular-convective pattern caused by surface-tension-driven instability under large temperature gradient was also an important physical phenomenon [58,59], these cellular convections in a melt pool had a very important impact on the crystal growth for the growing (moving) crystal interface can react to flow oscillations and can incorporate them as solidification microstructures [60].

The temperature gradients in SLM pools were asymmetrical and inclined to the free surface, coexistences of both vertical and horizontal gradients can be considered [61,62]. The vertical Marangoni number and horizontal Marangoni number are defined:

(2) $$Ma_{ver}=\left|\frac{\partial \sigma }{\partial T}\right|\Delta T_{ver}d\eta^{-1}\chi^{-1}$$

(3) $$Ma_{hor}=\left|\frac{\partial \sigma }{\partial T}\right|grad_{x}T_{}d^{2}\eta^{-1}\chi^{-1},$$

where Δ*T_ver_* is the vertical temperature gradient; *grad_x_T* is the horizontal temperature gradient; *d* is the layer thickness or length of the instability (1 × 10^−6^ m in vertical, 75 × 10^−6^ m in horizontal, as discussed below); *η* is the dynamic viscosity (6.44 mPa·s); *χ* is the thermal diffusivity (1.89 × 10^−5^ m^2^·s^−1^); *∂*σ/*∂*T = −0.39 × 10^−3^ N·m^−1^·K^−1^. Thus, the vertical Marangoni number M*a_ver_* (near the bottom and in front of S/L interface) can be estimated roughly as 2000 and the horizontal M*a_hor_* (near the laser melt track edge) was roughly about 180. It is known that when a liquid layer had a vertical temperature gradient and the M*a_ver_* becomes higher than the critical one (Δ*T* becomes higher than the critical Δ*T_c_*), the so-called Bénard–Marangoni-instability occurs in the form of hexagonal cells driven by surface tension [59,63,64,65]. The M*a_ver_* and M*a_hor_* calculated above are remarkably large and the instability is inevitable. When the M*a_ver_* is predominant and vertical melt flow has priority, the flow patterns have cellular structures (Bénard-Marangoni drifting cells and surface drifting cells). When the M*a_hor_* was predominant and horizontal melt flow had the priority, the flow patterns had roll/strip structures (longitudinal rolls and surface longitudinal rolls). The vertical cells (vertical instability) can be generated in a very thin layer (1 × 10^−6^ m) but the horizontal rolls (horizontal instability) needed a longer surface convective region. As illustrated earlier the cell spacing was around 1 μm, but the strips has a length over 50 μm, as in Figure 2 and Figure 3. Transitions between different convective patterns occurring by changing the governing parameter *Ma* have been proved in [64]. Another similar case was the electron beam melting of high melting point metals, the beam heats the free surface of the melt and simultaneous radiative cooling is very significant, resulting in drifting cellular structures on the melt surface [61].

Based on the stability limit analysis and flow pattern transitions, the cellular sub-grain microstructure shown in Figure 5c can be attributed to the Bénard-Marangoni-instability. In a very thin layer (1 × 10^−6^ m) in front of the S/L interface and at the melt pool bottom, there exists a very strong temperature gradient [66]. Thereby the convection with hexagonal structures can be generated in this thin layer and be superimposed on grain solidification at the same time. In conclusion, the solidification mode and morphologies of macro-grains were controlled by *G* and *R*, the sub-grain patterns are controlled by flow instabilities and M*a*, these two mechanisms are combined. Moreover, by the variations of fluid flow condition and the weld pool oscillation, the ideal hexagonal structures lose its stability to other geometries. Individual hexagons undergo local changes in topology and transform first into pentagons and then into squares [67], so the sub-grain patterns within the macro-grain can be a mixture of hexagons, pentagons and squares. In addition, this instability can always be generated in front of the S/L interface, and advances accompany with the S/L interface movements until the sub-grain patterns are created in each of the macro-grains in the bulk.

**Figure 9:**
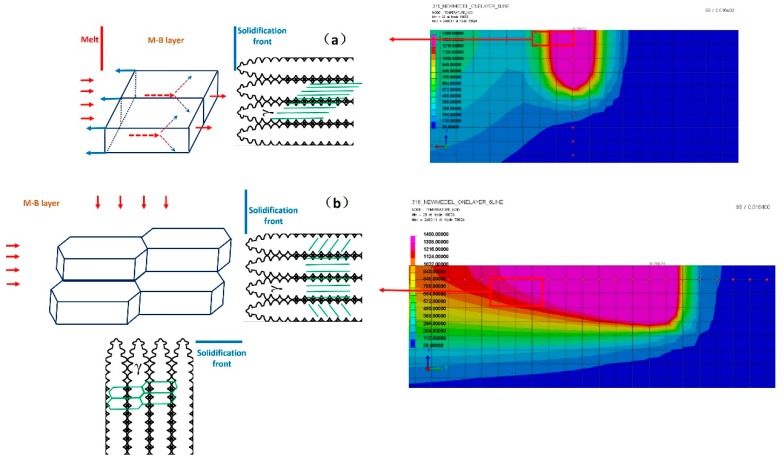
The forming mechanism of the bands in (**a**) and mixed patterns with cellular, elongated cellular and bands structures in (**b**).

**Figure 10:**
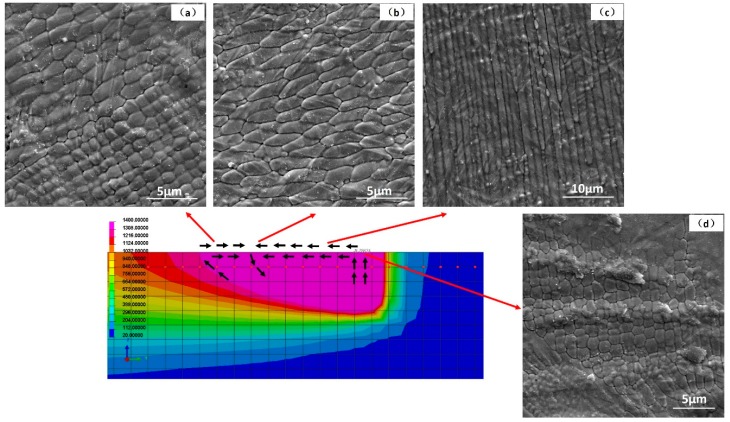
The forming mechanism of as-melted top surface morphologies, (**a**) mixture of cellular and elongated cellular, (**b**) elongated cellular, (**c**) strips and (**d**) quasi-hexagonal cellular structures.

In another situation, when a strong horizontal temperature gradient and horizontal fluid flow exist in the melt edge area, streak structures can be generated from melt pool edge to the center, as in Figure 9a. These streak structures can also be superimposed on macro-grain solidification and the intragranular band patterns are generated, as Figure 5f. Ideal conditions exist for structures, as in Figure 9a, but the actual temperature gradients in SLM pools are asymmetrical and complicated. These interactions can generate more complex sub-grain patterns, as in Figure 9b. The cellular, elongated cellular and bands appear simultaneously and transitionally (Figure 5). The as-melted top surface morphologies can also be explained by this mechanism and these morphologies are generated in the last stage of solidification, reflecting the complex surface Marangoni flows as plotted in Figure 10.

### 4.3. The Micro-Segregations of Sub-Grain Cellular Structures

The thermocapillary flow and Bénard–Marangoni-instabilities in front of the S/L interface can also be proved by the inclusions and micro-segregations in SLM sub-grain patterns of 316 L and of the Al–Si alloy. The equivalents of Cr (*Cr_eq_*) and Ni (*Ni_eq_*) of the precursor powders, of the SLM cellular microstructure and of the Cr–Si–O inclusions were calculated according to Schaeffler predictive phase diagram [68]. For the precursor powder, it achieves 19.9 for *Cr_eq_* and 15.89 for *Ni_eq_*, with *Cr_eq_ = (Cr + Mo + 1.5Si + 0.5Nb)* and *Ni_eq_ = (Ni + 30C + 0.5 Mn)*. For the SLM sub-grain cellular microstructure, it attained 20.035 for *Cr_eq_* and 16.11 for *Ni_eq_*. Finally, for Cr–Si–O inclusions it was 32.95 for *Cr_eq_* and 15.84 for *Ni_eq_* (referred in [6]). The elemental composition of SLM cellular substructure was similar to the precursor powders, *Cr_eq_/Ni_eq_* is 1.25 for powders and 1.24 for SLM sub-grain cells. Comparing the Cr-Si-O inclusions with the precursor powders, austenite promoting elements (Ni, Mo) are depleted but ferrite promoting elements (Si, Cr, Ti) are increased, *Cr_eq_/Ni_eq_* is 2.08 for Cr–Si–O inclusions. According to the Schaeffler diagram and the WRC-1992 diagram, a single phase austenite will be formed by lower *Cr_eq_/Ni_eq_* (<1.37) and “austenite + acicular ferrite” will be generated for higher *Cr_eq_/Ni_eq_* (≈2) [29,68]. That means 316 L steel solidifies with a single-phase austenitic microstructure during SLM, which consumes the austenite-promoting Ni, and rejects the ferrite-promoting elements Cr, Si and Mo in the solidification front, thus the ratio of *Cr_eq_/Ni_eq_* increases ahead of the S/L interface.

Another physical phenomenon named particle accumulation structures (PAS) will be introduced [66,69,70,71]. PAS is the behavior of small particles of dilute concentration in a time-dependent (oscillatory) vortex thermocapillary flow. The evenly distributed particles will form clouds circulating in the vortex when affected by an oscillatory thermocapillary flow [69,70,71]. Effect of PAS has an impact on the discussion of SLM, where the austenitic solidification of 316 L steel will reject the ferrite-promoting elements Cr, Si and Mo. The two elements Cr and Mo increase the local surface tension (solutocapillary) and Si increases the melt liquidity. The enhanced capillary vortex will bring the rejected ferrite-promoting elements Cr, Si and Mo from the S/L interface to the instability layer. The elements Si and Cr have strong affinity to oxygen and will react with residual oxygen and form the mentioned Cr–Si–O inclusions. These will precipitate together with the Mo-enrichment at the cellular boundaries by Marangoni–Bénard convection and PAS mechanism. For the higher *Cr_eq_/Ni_eq_* at the boundary, it changes the solidification mode from “full austenitic” to “austenite + acicular ferrite”. Then the solid-state phase transformation occurs during cooling, from ferrite to austenite with composition invariant massive reaction, that is the main reason of high dislocation concentrations observed and it contributes to the resistance toward an acid etching agent at the sub-grain boundary. 

These explanations can also be used for structures found in an Al–Si eutectic formed by SLM. The microstructure is significantly different when compared with a casted Al–Si alloy. A continuous eutectic structure of Al and Si is displayed along with dispersed primary α-Al in casted Al-Si alloy [17], whereas the Al-Si SLM microstructure consists of cellular morphologies. EDS analysis revealed that Si was preferentially located at the cellular boundaries (thickness of about 200 nm) and the cells of 500–1000 nm size were richer in Al. Based on the Al–Si phase diagram, the solubility of Si in Al was 1.65 wt.% at 850 K but decreased to 0.06 wt.% at 573 K [16]. Thus, the solidifying front rejects Si ahead of the S/L interface. Under the mentioned convection and PAS mechanism, the rejected Si particles will be redistributed by the intense capillary flow. Similar patterns as seen in SLM 316 L (cellular, elongated cellular, bands) were preferred and residual Si micro-segregations were seen along such boundaries. This mechanism can also be tested by the results of different base plate heating’s, the microstructures obtained with base plate heating and without base plate heating have some differences, the width of dendrites increase, and laminar eutectic appears with base plate heating. [18]. That could due to the reduced Δ*T* and driving force of convections, giving a tendency to solidify more as the eutectic casting.

## 5. Conclusions

(1) In this paper, the SLM alloys fine sub-grain cellular/bands microstructures are found have some natural transitions and morphology evolutions from quasi-hexagonal-cells to elongated cells/bands in the whole SLM bulk. The cellular, elongated cellular and bands structures can coexist inside one single macro-solidified grain with size around 1 μm.

(2) The sub-grain patterns are created by the couple effects of solid/liquid interface instability and melt hydrodynamic instability (Bénard instabilities). The nonlinear self-organization phenomenon is superimposed on the macro-grain solidification to form the sub-grain patterns and microsegregations.

(3) The austenitic solidification of 316 L steel will reject the ferrite-promoting elements Cr, Si and Mo at the S/L interface and the enhanced capillary vortex will bring the rejected elements from the instability layer to the melt. The two elements Si and Cr will react with residual oxygen and form the precipitated Cr-Si-O inclusions and the element Mo will enrich at the sub-grain cellular boundaries. These explanations can also be used to explain the sub-grain microstructures in Al–Si and Co–Cr–Mo SLM, Si and Cr, Mo microsegregate in the FCC prevenient phase sub-grain cellular boundaries separately (Al and Co-rich γ phase).

(4) Currently this phenomenon is only obvious in the eutectic alloys with the FCC prevenient phase, which could relate to the solid-liquid two-phase lines distance and mushy zones, together with FCC atom interstitial positions and impurity’s solubility. Broader differences between solidus/liquidus temperature prone to activate the S/L interface instabilities and planar interface bifurcating into cellular microstructure. Limited by the article length and topic focus, this section is not included in this paper but becoming an article unto itself.

This knowledge can be further used to manipulate the SLM microstructures, enhance the as-fabricated mechanical and physical properties.
